# Experience with *Saccharomyces boulardii* Probiotic in Oncohaematological Patients

**DOI:** 10.1007/s12602-017-9332-4

**Published:** 2017-09-25

**Authors:** Beata Sulik-Tyszka, Emilian Snarski, Magda Niedźwiedzka, Małgorzata Augustyniak, Thorvald Nilsen Myhre, Anna Kacprzyk, Ewa Swoboda-Kopeć, Marta Roszkowska, Jadwiga Dwilewicz-Trojaczek, Wiesław Wiktor Jędrzejczak, Marta Wróblewska

**Affiliations:** 1 Department of Microbiology, Central Clinical Hospital in Warsaw, Warsaw, Poland; 2 Department of Haematology, Oncology and Internal Diseases, Medical University of Warsaw, Warsaw, Poland; 3 Hospital Pharmacy, Central Clinical Hospital in Warsaw, Warsaw, Poland; 4 Department of Dental Microbiology, Medical University of Warsaw, 1a Banacha Street, 02-097 Warsaw, Poland

**Keywords:** *Saccharomyces boulardii*, Probiotic, Leukaemia, Lymphoma, Haematology

## Abstract

Very few reports have been published to date on the bloodstream infections caused by *Saccharomyces* spp. in oncohaematological patients, and there are no guidelines on the use of this probiotic microorganism in this population. We describe the use of probiotic preparation containing *Saccharomyces boulardii* in a large group of oncohaematological patients. We retrospectively analysed the data from 32,000 patient hospitalisations at the haematological centre during 2011–2013 (including 196 haematopoietic stem cell transplant recipients) in a tertiary care university-affiliated hospital. During the study period, 2270 doses of *Saccharomyces boulardii* probiotic were administered to the oncohaematological patients. In total, 2816 mycological cultures were performed, out of which 772 (27.4%) were positive, with 52 indicating digestive tract colonisation by *Saccharomyces* spp., mainly in patients with acute myeloid leukaemia (AML), myelodysplastic syndrome (MDS) or multiple myeloma (MM). While colonised, they were hospitalised for 1683 days and 416 microbiological cultures of their clinical samples were performed. In the studied group of patients, there were six blood cultures positive for fungi; however, they comprised *Candida* species: two *C. glabrata*, one *C. albicans*, one *C. krusei*, one *C. tropicalis* and one *C. parapsilosis*. There was no blood culture positive for *Saccharomyces* spp. Our study indicates that despite colonisation of many oncohaematological patients with *Saccharomyces* spp., there were no cases of fungal sepsis caused by this species.

## Introduction

*Saccharomyces boulardii* is a fungus classified as a yeast, which routinely cannot be distinguished from *Saccharomyces cerevisiae* and at present is regarded as a subtype of *S. cerevisiae* [1–6]. These yeasts are widespread in nature and can be found on plants, fruit and in soil, being also used in the baking and brewing industry [1, 2]. Usually, they are considered to be nonpathogenic commensals of the digestive tract, administered as probiotics for several indications, including *Clostridium difficile*-associated disease [4, 7–16].

However, since the 1990s, an increasing number of publications of fungemia and invasive infections caused by *Saccharomyces cerevisiae* (*boulardii*), particularly among intensive care unit (ICU) patients and individuals with multiple co-morbidities, have been reported in literature in patients treated with a probiotic preparation containing this yeast [1, 2, 5, 6, 11, 17–21]. Furthermore, it can also cause infections in otherwise healthy individuals [13, 17, 22–24].

Diarrhoea and colonisation of the gastrointestinal tract by potentially pathogenic bacteria including *Clostridium difficile* are important problems in care of oncohaematological patients treated for leukaemia and lymphoma [25]. One of the possible approaches to this problem is to use probiotics containing *Saccharomyces boulardii* [26]. Both McFarland, and Videlock and Cremonini in their meta-analyses comprising 5029 patients and 4138 patients, respectively, concluded that probiotic preparations containing *Saccharomyces boulardii* reduced the incidence of antibiotic-associated diarrhoea (AAD) [8, 27].

However, there are concerns that probiotics might lead to severe invasive infections in some patients. This notion is supported by some case reports of *S. boulardii* or *S. cerevisiae* sepsis [1, 9, 18, 28]. This risk is emphasised in guidelines for infection prevention in haematological patients, in which probiotics usage is generally contraindicated or their possible use is not mentioned at all [29, 30]. Oral administration of probiotic preparation containing live yeasts may pose a particularly high risk to oncohaematological patients as they often suffer from severe immune deficiency due to malignancy [29]. Furthermore, immunosuppressive treatment itself predisposes these patients to infections and increases the risk of spread of microorganisms within the host. It should also be noted that oral mucositis and ulcers are very common in oncohaematological patients. It may lead to yeast translocation through the oral mucous membrane into the bloodstream, causing fungaemia and invasive infections linked to an increased mortality in this group of patients.

However, the basis of the above-mentioned guidelines relies on a limited number of published cases, and there is hardly any evidence from clinical trials to support this belief. The guidelines are based on two reports published in 1996. In one of them, 55 cases of *Lactobacillus* infections were reviewed—the mortality rate for the entire group was 6%; however, there were no deaths among the patients with acute myeloid leukaemia (AML) (*n* = 1), HIV (*n* = 3) and neutropenic fever (*n* = 3) [31]. In the other publication, a case was reported of a patient who developed *Mucor* spp. infection 5 months after haematopoietic stem cell transplantation (HSCT) and was using an excess of oral herbal remedies with purified reishi mushrooms, extracts of ginseng and seaweed, and concentrated preparation of *Lactobacillus* bacteria [32]. Apart from the guidelines, there is at least one publication referring to a larger cohort of patients with sepsis caused by *Saccharomyces boulardii* (*n* = 37) [1]. In this publication, there was only one patient with AML and four patients with HIV infection in whom sepsis caused by this pathogen was confirmed—in all cases with favourable outcomes.

In this study, we report our experience with a wide use of a probiotic preparation containing *Saccharomyces boulardii* in patients with haematological neoplasms.

## Patients and Methods

The study is a retrospective analysis of the hospital and medical data of the patients who were treated at the haematology and oncology ward from January 2011 until the end of December 2013 in a tertiary care university-affiliated teaching hospital (1050 beds).

The data from the microbiology department was used to assess the total number of microbiological cultures performed in the haematology and oncology department in the study period and find all of the patients who were colonised with fungi or had invasive fungal infections. Furthermore, we analysed the medical charts of all the patients who were colonised with *Saccharomyces boulardii* in order to assess their use of probiotics and antibiotics, the occurrence of colonisation and invasive infections caused by this yeast, as well as occurrence of neutropenia and previous treatment regimens used in this group of recently treated immunocompromised patients. Moreover, the data of the hospital pharmacy were analysed to assess the total use of probiotics in the haematology and oncology department.

The Enterol 250 (Biocodex, France) was used as a probiotic preparation, containing 250 mg of lyophilised *Saccharomyces boulardii* in each tablet.

Specimens for microbiological cultures were taken from the patients as a standard procedure, according to the following indications: a clinical suspicion of infection (fever > 38 °C or other signs of infection), diarrhoea and when a patient was admitted for a high-toxicity treatment. As a routine procedure, surveillance oral and rectal swabs were taken. The patients who received probiotics were treated according to the same protocol as other patients and did not undergo any additional surveillance.

Mycological cultures of clinical specimens and identification of strains of *Saccharomyces* spp. were done according to the standard laboratory procedures. Clinical samples were inoculated onto Sabouraud agar supplemented with chloramphenicol and gentamycin (bioMerieux), and incubated aerobically at 30 °C for 10 days. Identification of the strains of *Saccharomyces* spp. was done using API ID 32 C (bioMerieux) tests and matrix-assisted laser desorption-ionisation time of flight mass spectrometry (MALDI-TOF MS) (MALDI Microflex LT, Bruker, Germany).

## Results

During the study period (January 2011–December 2013), there were 32,000 hospitalisations of haematological patients (including hospitalisations of 116 autologous and 82 allogeneic transplant recipients and daycare unit stays). During this period, the only probiotic administered to the patients in the haematology and oncology department was preparation containing *Saccharomyces boulardii*. The probiotic was given to the patients once daily in the dose of 250 mg of lyophilised *Saccharomyces boulardii*. In total, during the study period, 2270 doses of the preparation were administered to the patients on the ward (Table 1).

**Table 1:** Use of the probiotics containing *Saccharomyces boulardii*, occurrence of colonisation with this microorganism and invasive infections in oncohaematological patients

Number of doses of the probiotic to the patients during the study	Number of patients with colonisation of the digestive tract (detected in oral swabs or stool samples)	Total number of days of observation of colonised patients	Number of mycological cultures performed in the study period in hospitalised oncohaematological patients	Number of invasive infections by *Saccharomyces* spp. (sepsis or systemic infection)
2270	38	1683	2816	0

The cultures of oral swabs and stool samples were performed for various indications

During the study, 2816 mycological cultures of the specimens, collected from patients hospitalised in the department of haematology and oncology, were performed. There were 772 positive fungal cultures, comprising 48 rectal swab and 5 oral swab cultures positive for *Saccharomyces boulardii*, obtained from 38 patients during the study period. There were six blood cultures positive for fungi; however, they comprised *Candida* species: two *C. glabrata*, one *C. albicans*, one *C. krusei*, one *C. tropicalis* and one *C. parapsilosis*. In the studied group of patients, there was no blood culture positive for *Saccharomyces* spp. To put this data into the perspective, there were 22 blood cultures positive for *Saccharomyces boulardii* in patients hospitalised in other wards of the hospital—in internal medicine wards (3 cases), in surgery wards (6 cases) and in the intensive care unit (13 cases).

The basic demographic data of the population of colonised oncohaematological patients are shown in Table 2. The group of colonised patients consisted predominantly of patients with AML, MDS or MM. Colonisation of these patients, detected in the surveillance swabs taken during the whole hospitalisation period, lasted on average for 1–4 weeks, rarely—for up to 3 months. They received the median of three chemotherapy cycles (range 0–16) prior to colonisation, and while colonised, they were hospitalised for 1683 days, with 416 microbiological cultures performed. We have analysed the use of probiotic in 23 of them for the median of 27 days (range 6–69 days) accounting for 1019 doses of the preparation (45% of the probiotics used in the ward during the study period). The probiotics were given due to diarrhoea (10 out of 23) or colonisation with *Clostridium difficile* with or without diarrhoea (10 out of 23). As mentioned above, we did not identify any invasive infections with *Saccharomyces boulardii* or with *Saccharomyces cerevisiae* in this group of patients.

**Table 2:** General data of the colonised patients included in the study

Characteristics	Colonised patients (*n* = 38) and number of hospitalisations (*n* = 53)
Median age on admission in years (range)	61 (19–81)
Median hospital stay in days (range)	36 (2–112)
Gender, *n* (%)	
Female	20 (*53*)
Male	18 (*47*)
Disease, *n* (%)	
Acute myeloid leukaemia	11 (*29*)
Myelodysplastic syndromes	7 (*18*)
Multiple myeloma	7 (*18*)
Lymphoma	4 (*11*)
Chronic myelo-monocytic leukaemia	2 (*5*)
Acute lymphoblastic leukaemia	2 (*5*)
Other	5 (*14*)
Cause of hospitalisation, *n* (%)	
Chemotherapy	26 (*68*)
Haematopoietic stem cell transplantation	5 (*14*)
Other	7 (*18*)
Probiotic use, *n* (%)	26 (*72*)
Duration of use of probiotic in days (range)	27 (6–69)
Indication for *Saccharomyces boulardii* probiotic, *n* (%)	
Diarrhoea	10 (43)
*Clostridium difficile* (with or without diarrhoea)	10 (43)
Unknown	3 (14)
Median number of microbiological cultures per patient during hospitalisation (range)	8 (1–41)
Median number of mycological stool cultures per patient during hospitalisation (range)	3 (0–8)
Neutropaenia during *S. boulardii* colonisation, *n* (%)	14 (36)
Antibiotic treatment at the time of *S. boulardii* colonisation, *n* (%)	38 (*97*)
Median number of antibiotics used during hospitalisation (range)	5 (0–10)
Antifungal prophylaxis/treatment at the time of *S. boulardii* colonisation, *n* (%)	34 (*89*)
Presence of the central venous catheter, *n* (%)	26 (*63*)
Parenteral nutrition at any time during hospitalisation, *n* (%)	7 (*18*)
Fever during hospitalisation, *n* (%)	26 (*74*)
Body mass index (BMI) < 18.5, *n* (%)	7 (*21*)

As some medical records were not complete, the percentages given in italics are the percentage of data from complete records of this parameter

## Discussion

Probiotics have been used safely for years, and at present, they are indicated for therapy or prophylaxis of many diseases, such as enteral nutrition-related diarrhoea; prevention of *C. difficile*-associated disease (CDAD) and recurrences of this condition; treatment of irritable bowel syndrome, traveller’s diarrhoea and Crohn’s disease; and reduction of *Helicobacter pylori* treatment-related symptoms, giardiasis and human immunodeficiency virus-related diarrhoea, as well as to preserve the intestinal bacterial flora during antibiotic therapy [1, 4, 8–16, 26, 33]. Their mechanism of action is not fully understood, but it has been claimed that *S. boulardii* may directly interact and inhibit *C. difficile* toxin A [15]. However, their safety—particularly in imunocompromised patients—remains a controversial issue [1, 7, 16]. Several authors emphasise that caution is necessary when administering probiotics to the patients with impaired immunity and risk-benefit potential should be evaluated [2, 5, 6, 10, 12, 32]. This is particularly important while administering probiotics containing yeasts to the immunocompromised patients [34]. Damage to the mucosal barriers (e.g. mucositis and oral ulcers often seen in oncohaematological patients) as well as immunosuppression due to the underlying condition or administered treatment (apart from other risk factors) may contribute to invasive fungal infections in this group of patients.

To our knowledge, this is the first report of a wide usage of probiotics in oncohaematological patients. Interestingly, although the use of this probiotic led in some patients to colonisation of the digestive tract with this yeast, there was no single case of sepsis caused by this microorganism in this high-risk population.

We have identified patients colonised with *S. boulardii* and analysed their probiotic use, which accounts for about half of the use of probiotics in the ward. As this population was treated with chemotherapy courses and had neutropaenia for prolonged periods of time, it seems that *S. boulardii* has only a limited ability to cause sepsis in this patient population despite severity of their clinical condition. The main indication for *Saccharomyces boulardii* use was diarrhoea or colonisation with *Clostridium difficile*. However, with the study design, we did not assess the effectiveness of the probiotic preparation for this indication.

Despite the fact that oncohaematological patients in the studied population were intensively treated with antibiotics (97%) and antifungals (90%) during the period of colonisation with *Saccharomyces boulardii*, we did not notice any effect of this treatment on occurrence of invasive infections in this population, although invasive *Saccharomyces boulardii* infections were recorded in patients hospitalised in other wards of the hospital during the study period. Salonen et al. in a study involving patients with haematological diseases reported isolation of *S. cerevisiae* from throat, stool, urine, and perineum samples in 16, 23, 10 and 20% of the patients, respectively [35]. Interestingly, in another study, among 70 patients hospitalised for bone marrow transplantation, *S. cerevisiae* was isolated only once, despite weekly surveillance cultures (primarily of stool and throat swab specimens) during hospitalisation [36]. It is possible that antifungal treatment or prophylaxis—administered as a routine regimen to oncohaematological patients—prevented in our patients progression of *S. boulardii* colonisation to an invasive infection. In empiric therapy in these patients, voriconazole is used, which is active against *Aspergillus* spp., *Candida krusei* and *Candida glabrata*, and it may have also prevented invasive infections of *S. boulardii*/*S. cerevisiae* aetiology. Indeed, Burkhardt et al. reported a successful treatment of *S* ***.*** *boulardii* sepsis with voriconazole, while initial treatment with fluconazole was not effective [9].

It appears that *S. boulardii*/*S. cerevisiae* may spread on the ward causing an outbreak among hospitalised patients [2, 19, 37]. It should be emphasised that more stringent isolation procedures and other infection control precautions in the haematology/oncology ward diminish the risk of spread of this and other microorganisms among oncohaematological patients. Reports in literature indicate that risk factors predisposing to *S. boulardii* infections are very similar to those of invasive candidiasis, and clinically, these infections are indistinguishable [1]. It is therefore very important to prevent healthcare-associated infections in these patients, including central venous catheter-related bloodstream infections, as *S. boulardii* very often causes fungaemia [1, 2, 19, 38–42]. Several authors indicate the need for proper handling of this preparation during its administration [19, 39]. It has been postulated that further studies are needed to evaluate the efficacy of probiotic preparations, to determine the optimal dose and strain of probiotic, and possible adverse events related to their use [7, 14, 32].

This is the first report of an experience with a wide use of probiotics in oncohaematological patients showing no invasive infection or sepsis caused by this microorganism in a large population of patients with impaired immunity. The results of this study justify further studies on the use of probiotics and their safety in oncohaematological patients, as well as evaluation of their efficacy in patients with diarrhoea after chemotherapy, colonised with *Clostridium difficile* or multi-drug-resistant strains of bacteria, patients with neutropaenia and haematopoietic stem cell transplant recipients.
